# Relationship between Penicillin-Binding Proteins Alterations and β-Lactams Non-Susceptibility of Diseased Pig-Isolated *Streptococcus suis*

**DOI:** 10.3390/antibiotics12010158

**Published:** 2023-01-12

**Authors:** Kamonwan Lunha, Wiyada Chumpol, Surasak Jiemsup, Sukuma Samngamnim, Pornchalit Assavacheep, Suganya Yongkiettrakul

**Affiliations:** 1 National Center for Genetic Engineering and Biotechnology, National Science and Technology Development Agency, Pathum Thani 12120, Thailand; 2 Department of Veterinary Medicine, Faculty of Veterinary Science, Chulalongkorn University, Bangkok 10330, Thailand

**Keywords:** β-lactams resistance, penicillin resistance, penicillin-binding proteins, *Streptococcus suis*

## Abstract

*Streptococcus suis* is a zoonotic pathogen causing disease in both animals and humans, and the emergence of increasingly resistant bacteria to antimicrobial agents has become a significant challenge globally. The objective of this study was to investigate the genetic basis for declining susceptibility to penicillin and other β-lactams among *S. suis*. Antimicrobial susceptibility testing and penicillin-binding proteins (PBP1a, PBP2a, PBP2b, and PBP2x) sequence analysis were performed on 225 *S. suis* isolated from diseased pigs. This study found that a growing trend of isolates displayed reduced susceptibility to β-lactams including penicillin, ampicillin, amoxicillin/clavulanic acid, and cephalosporins. A total of 342 substitutions within the transpeptidase domain of four PBPs were identified, of which 18 substitutions were most statistically associated with reduced β-lactams susceptibility. Almost all the *S. suis* isolates which exhibited penicillin-non-susceptible phenotype (71.9%) had single nucleotide polymorphisms, leading to alterations of PBP1a (P409T) and PBP2a (T584A and H588Y). The isolates may manifest a higher level of penicillin resistance by additional mutation of M341I in the ^339^STMK active site motif of PBP2x. The ampicillin-non-susceptible isolates shared the mutations in PBP1a (P409T) and PBP2a (T584A and H588Y) with additional alterations of PBP2b (T625R) and PBP2x (T467S). The substitutions, including PBP1a (M587S/T), PBP2a (M433T), PBP2b (I428L), and PBP2x (Q405E/K/L), appeared to play significant roles in mediating the reduction in amoxicillin/clavulanic acid susceptibility. Among the cephalosporins, specific mutations strongly associated with the decrease in cephalosporins susceptibility were observed for ceftiofur: PBP1a (S477D/G), PBP2a (E549Q and A568S), PBP2b (T625R), and PBP2x (Q453H). It is concluded that there was genetically widespread presence of PBPs substitutions associated with reduced susceptibility to β-lactam antibiotics.

## 1. Introduction

*Streptococcus suis* is one of the most common Gram-positive cocci that usually colonizes the upper respiratory tract of pigs and can cause respiratory and systemic diseases, particularly in the postweaning period. In addition, it is also a serious zoonotic infection, causing meningitis and toxic shock-like syndrome. Most of the *S. suis* infection cases occurred in Asian countries, especially in China, Vietnam, and Thailand [1]. 

Although autogenous vaccines are used in pig farms, they are serotype-specific and give inconsistent cross protection against heterogeneous *S. suis* [1]. Antimicrobials remain the critical treatment for *S. suis* infection. Antibiotic consumption is extensively used in the livestock sector, especially for pigs [2,3]. The most commonly used antibiotics in the swine industry are amoxicillin, enrofloxacin, tetracycline, and penicillin [3]. 

Major empirical antimicrobial drugs for treating *Streptococcus* spp. infection are β-lactams such as penicillin and cephalosporins, which target penicillin-binding proteins (PBPs), cell wall transpeptidase catalyzing the crosslink of bacterial cell wall peptidoglycan [4]. In PBPs, three conserved motifs, SXXK (with the active site serine), SXN, and KT(S)G found in transpeptidase domain (TPD) form the catalytic center. The β-lactams function by covalently binding to the active site serine through the β-lactam ring, thereby interfering with the synthesis of bacterial cell walls and leading to bacterial cell death [5,6]. However, *Streptococcus* pathogens with β-lactams resistance are continually found worldwide [4,5,7]. The main mechanism of β-lactams resistance is alterations in the transpeptidase of PBPs, leading to low affinity to β-lactams, which thus confers resistance [4,5,6]. 

As for *S. pneumoniae*, *S. suis* has four key PBPs including PBP1a, 2a, 2b, and 2x. However, while several studies have described the genetic profile of TPD of PBPs in association with β-lactams resistance in *S. pneumoniae*, only limited data are available on *S. suis* isolates [4,5,6]. A high degree of distinctiveness was observed over the different geographical areas, by taking into account the different environmental pressures, management of antibiotic prescription, and health conditions [1,8]. Therefore, more understanding of mutations within the PBPs and their relation to β-lactams resistance in *S. suis* isolates, specifically from an endemic area, is critical. The purpose of this study was to determine the relationship between specific TPD of PBPs alterations and β-lactams-non-susceptible in *S. suis* isolated from diseased pigs in Thailand.

## 2. Results

### 2.1. Characteristics of S. suis Isolates

*S. suis* 225 isolates were identified, based on ≥95% identity of specific recombination/repair protein (*recN*) nucleotide sequences and ≥94% average nucleotide identity (ANI) to the reference strain, *S. suis* P1/7, AM946016.1. The multiplex PCR reaction and WGS analysis differentiated 195 *S. suis* isolates with specific serotype and 30 isolates with non-typeable (NT) serotype. Among the 195 serotypeable isolates, 24 serotypes were identified. No *S. suis* isolates with serotype 6, 12, 13, 17, and 19 were found in this study. The top five serotypes were: 2 (25.8%, 58/225), 8 (8.4%, 19/225), 29 (7.6%, 17/225), 9 (6.2%, 14/225), and 3 (5.3%, 12/225). The serotypes 2 and 8 were major serotypes found in lung isolates, accounting for 25.0% (46/184) and 10.3% (19/184), respectively (Table S1).

### 2.2. Distribution of Minimum Inhibitory Concentrations

**Figure 1:**
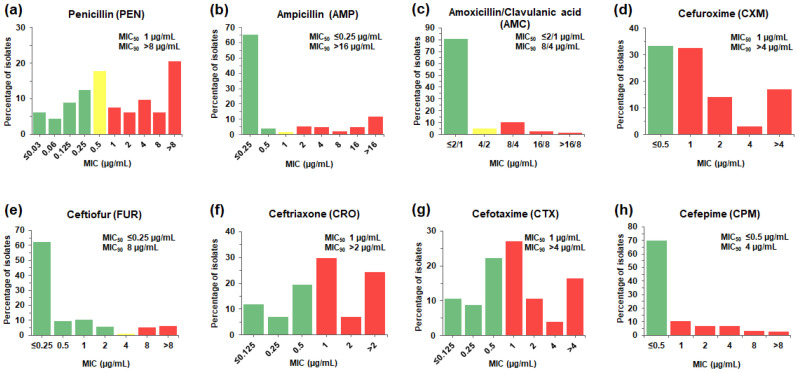
MIC distributions of β-lactam antibiotics for the 225 *S. suis* isolates. The frequencies of different MICs for each of the β-lactams tested were tabulated as a percentage of isolates (%) with a particular MIC value (µg/mL) for penicillin (**a**), ampicillin (**b**), amoxicillin/clavulanic acid (**c**), cefuroxime (**d**), ceftiofur (**e**), ceftriaxone (**f**), cefotaxime (**g**), and cefepime (**h**). The color of bar charts indicates interpretive range for each of the antibiotics; susceptible (green), intermediate (yellow), and resistant (red). MIC_50_ and MIC_90_, the MIC which inhibit 50% and 90% of the isolates tested, respectively, were also indicated.

The distribution of β-lactam MICs is shown in Figure 1 and Table S2. β-lactams-non-susceptible *S. suis* was defined by the MIC breakpoint values for the interpretation of resistance and intermediate susceptibility. The result showed that there were 68.0% (153/225) of the isolates classified as penicillin (PEN)-non-susceptible *S. suis* (MIC of 0.5–>8 µg/mL), including PEN-resistant *S. suis* with MIC of 1–>8 µg/mL (50.2%, 113/225) and PEN-intermediate *S. suis* with MIC of 0.5 µg/mL (17.8%, 40/225). The prevalence of amoxicillin/clavulanic acid (AMC) and ampicillin (AMP)-non-susceptible *S. suis* isolates (MIC of 4/2–>16/8 and 1–>16 µg/mL, respectively) was about 19.1% (43/225) and 30.7% (69/225), respectively.

The prevalence of non-susceptible *S. suis* isolates to the second-generation cephalosporins, cefuroxime (CXM) was 66.7% (150/225). Among the third-generation cephalosporins, *S. suis* exhibited non-susceptibility to ceftiofur (FUR) with the lowest prevalence (12.4%, 28/225), followed by cefotaxime (CTX) (58.2%, 131/225) and ceftriaxone (CRO) (61.3%, 138/225). Whereas the presence of isolates resistant to the fourth-generation cephalosporin, cefepime (CPM) (30.2%, 68/225), was found.

### 2.3. Alterations of Penicillin-Binding Proteins

**Figure 2:**
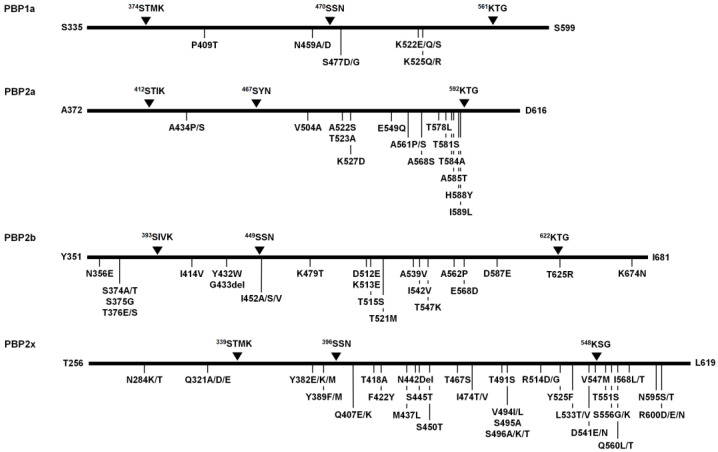
Schematic diagram displaying the conserved active site motifs of transpeptidase domain (TPD) in penicillin-binding proteins (PBPs) of *S. suis* and most common mutation sites observed among the 225 *S. suis* isolates. The conserved active site motifs are marked by black triangles. The alteration sites of PBPs with >40% prevalence are indicated below.

In this study, amino acid alterations of transpeptidase domain (TPD) of four penicillin-binding proteins (PBPs), PBP1a, PBP2a, PBP2b, and PBP2x, were observed from the 225 *S. suis* isolates. The TPD of PBP1a (position no. 335–599, 265 amino acids), PBP2a (position no. 372–616, 245 amino acids), PBP2b (position no. 351–681, 331 amino acids), and PBP2x (position no. 256–619, 364 amino acids) possess highly conserved active site motifs ^374^STMK˗^470^SSN˗^561^KTG, ^412^STIK˗^467^SYN˗^592^KTG, ^393^SIVK˗^449^SSN˗^622^KTG, and ^339^STMK˗^396^SSN˗^548^KSG, respectively (Figure 2). Compared with the *S. suis* reference strain P1/7 (AM946016.1), a total of 342 mutation sites (98, 35, 65, and 144 sites in PBP1a, PBP2a, PBP2b, and PBP2x, respectively) was identified within the TPD of all four PBPs. Throughout the entire TPD of individual PBPs, multiple amino acid alterations were observed at various positions, PBP1a (0–55 residues), PBP2a (0–22 residues), PBP2b (1–38 residues), and PBP2x (1–109 residues) (Table S3a–d, respectively).

According to the analysis of PBP1a of all *S. suis* isolates, the P409T was the most common substitution (54.7%, 123/225), followed by N459A/D, K522E/Q/S, and K525Q/R (44.9%, 101/225 each), and S477D/G (42.7%, 96/225) (Figure 2). Based on the distribution of various substitutions, the PBP1a alterations were classified into 27 different patterns of which the substitution rate was varied (0–20.8%). PBP1a type 1a-3 consisting of two amino acid substitution sites (E447A/D and A493S/T) was the most common alteration pattern (22.7%, 51/225) (Table S3a).

The analysis of PBP2a of all *S. suis* isolates demonstrated that the most common substitution was A561P/S, accounting for 97.8% (220/225) of the isolates, followed by T584A (55.1%, 124/225), H588Y (54.7%, 123/225), A434P/S (48.9%, 110/225), T523A, T581S, A585T (46.2%, 104/225 each), V504A, A522S, K527D, T578L, I589L (45.8%, 103/225 each), E549Q, and A568S (45.3%, 102/225 each) (Figure 2). There were 29 alteration patterns for PBP2a with 0–9.0% substitution rate. PBP2a type 2a-4 consisting of three amino acid alterations (V390I, A434P/S, and A561P/S) was the most common alteration pattern (21.3%, 48/225) (Table S3b).

Analysis of PBP2b revealed 62 substitution sites and 3 deletion sites along the 331 amino acids (positions 351 to 681) of TPD. The Y435del, was only found in one strain, whereas T671del and G433del were observed in 13.3% (30/225) and 76.0% (171/225) of *S. suis* isolates, respectively. The most common substitution was I414V (100%, 225/225), which was present in all the isolates, followed by Y432W, G433del (76.0%, 171/225 each), I452A/S/V (74.2%, 167/225), K479T, D512E, K513E, T515S (73.8%, 166/225 each), D587E (73.3%, 165/225), T625R (60.4%, 136/225), N356E (56.4%, 127/225), T376E/S (54.2%, 122/225), S374A/T (53.3%, 120/225), S375G (49.3%, 111/225), K674N (47.1%, 106/225), T521M, A562P (45.3%, 102/225 each), A539V, I542V, T547K (44.9%, 101/225 each), and E568D (43.6%, 98/225) (Figure 2). The PBP2b alterations found in all isolates were classified into 51 different PBP2b patterns with 0.3–11.5% substitution rate. Among them, PBP2b type 2b-16 consisting of 15 amino acid alterations was the most common alteration pattern (11.1%, 25/225) (Table S3c).

The analysis of PBP2x of all *S. suis* isolates revealed V494I/L was the most predominant (82.2%, 185/225), followed by Y382E/K/M (80.9%, 182/225), N284K/T, S450T, T551S, T491S (77.8%, 175/225 each), Y525F (76.9%, 173/225), R600D/E/N (76.4%, 172/225), Y389F/M (75.6%, 170/225), Q407E/K (75.1%, 169/225), N595S/T (74.7%, 168/225), T418A (73.8%, 166/225), D541E/N, S556G/K (71.1%, 160/225 each), V547M (69.8%, 157/225), I568L/T (64.0%, 144/225), D601S/T (62.7%, 141/225), F422Y (61.8%, 139/225), S496A/K/T (56.0%, 126/225), R288K, Q560L/T (54.2%, 122/225 each), Q321A/D/E, I474T/V (53.3%, 120/225 each), S495A (49.3%, 111/225), M437L, S445T (48.9%, 110/225 each), R514D/G, L533T/V (45.3%, 102/225 each), and T467S (42.2%, 95/225) (Figure 2). In addition, N442Del was observed in almost all *S. suis* isolates (99.6%, 224/225). According to the distribution of various substitutions in PBP2x, all isolates could be differentiated into 59 types with 0.3–29.9% substitution rate. The most common alteration pattern of PBP2x was type 2x-25 (22.7%, 51/225) consisting of 27 substitution sites (Table S3d).

### 2.4. Impact of PBP Substitutions on β-Lactams Susceptibility

**Figure 3:**
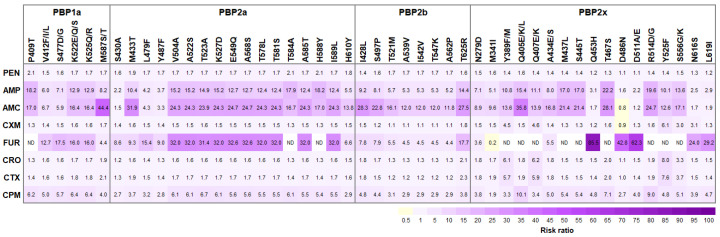
Risk ratio (RR) between candidate PBPs substitutions and β-lactams-non-susceptible phenotypes. Note only those 47 candidate substitutions with significantly differentiated β-lactams-susceptible and β-lactams-non-susceptible isolates (>50%) and found in β-lactams-non-susceptible *S. suis* isolates with high frequency (>70%), and M341I in ^339^STMK motif of PBP2x, are listed. Positive associations (RR > 1) are visualized in purple blocks and negative associations (RR < 1) in yellow blocks. Color intensity of the text labels represents proportional to the risk ratio. AMC, Amoxicillin/Clavulanic acid; AMP, Ampicillin; CPM, Cefepime; CRO, Ceftriaxone; CTX, Cefotaxime; CXM, cefuroxime; FUR, Ceftiofur; PEN, Penicillin. ND, the risk ratio analysis could not be determined, due to none of the FUR-non-susceptible *S. suis* isolates without PBP substitutions.

**Figure 4:**
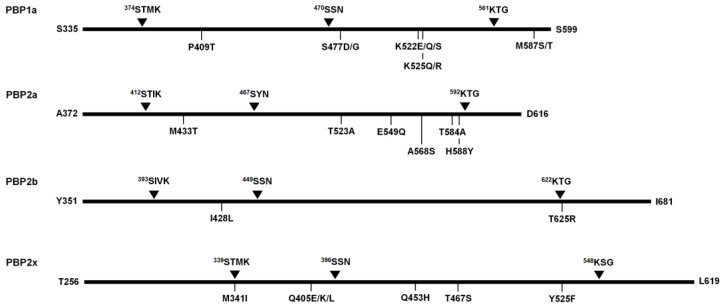
Schematic diagram presenting the major substitution sites implicated in β-lactams-non-susceptibility.

In an attempt to identify the impact of PBP substitutions on β-lactams susceptibility of *S. suis*, this study focused on the PBP substitutions that significantly differentiated between β-lactams-susceptible and β-lactams-non-susceptible isolates (>50%) and only found in β-lactams-non-susceptible *S. suis* isolates with high frequency (>70%). A total of 47 candidate residues were selected, including PBP1a (6 substitutions), PBP2a (17 substitutions), PBP2b (8 substitutions), and PBP2x (16 substitutions), that were postulated to mediate the decrease in β-lactams susceptibility. It is important to note that the PBP2x M341I was included in this study, due to the significance of the mutation in ^339^STMK active site motif (Figure 3 and Table S4). The relationship of all 48 selected candidate residues to β-lactams-non-susceptible phenotype was interrogated by risk ratio analysis. There were 18 amino acid substitutions with the highest risk ratio, exhibiting significant association with reduced β-lactams susceptibility (Figure 4). 

Among the PEN-non-susceptible isolates, an increase in RR was observed for PBP1a P409T, PBP2a P584A, and PBP2a P588Y, while amino acid substitutions in PBP2b and PBP2x exhibited low RR (<2.0). Similar substitutions in PBP1a (P409T) and PBP2a (P584A and P588Y) were also associated with the decrease in AMP susceptibility. Moreover, PBP2b T625R and PBP2x T467S exhibiting high RR (14 and 22, respectively) were found in AMP-non-susceptible isolates. The relationship between PBP substitutions, PBP1a M587S/T, PBP2a M433T, PBP2b I428L, and PBP2x Q405E/K/L, and AMC-non-susceptible isolates was demonstrated with the highest RR (44.4, 31.9, 28.3, and 35.8, respectively) (Figure 3 and Table S5). 

In this study, the strongest association between PBP substitutions and the decrease in cephalosporins susceptibility was reported. Only one substitution, PBP2x Y525F was significantly associated with CXM-non-susceptibility in *S. suis* (RR = 6.1). Five PBP substitutions, PBP1a S477D/G, PBP2a E549Q, PBP2a A568S, PBP2b T625R, and PBP2x Q453H, significantly contributed to the decrease in FUR susceptibility. CRO-non-susceptibility of *S. suis* isolates notably harboring PBP2b T625R and PBP2x Y525F. The PBP1a M587S/T, PBP2b T625R, and PBP2x Y525F substitutions were significantly associated with CTX-non-susceptibility of *S. suis* isolates. Several PBP substitutions, PBP1a K522E/Q/S, PBP1a K525Q/R, PBP2a T523A, PBP2b I428L, and PBP2x Q405E/K/L significantly contributed to the decrease in CPM susceptibility of *S. suis* isolates. In this study, the risk ratio analysis could not be conducted for the association between PBP substitutions, PBP1a (P409T), PBP2a (T584A and H588Y), and PBP2x (Y389F/M, Q405E/K/L, Q407E/K, M437L, S445T, T467S, R514D/G, Y525F, and S556G/K), and FUR-non-susceptible *S. suis*, due to none of the FUR-non-susceptibility of *S. suis* isolates without PBP substitutions (Figure 3 and Table S5).

## 3. Discussion

*S. suis* infection has been shown to have a significant economic loss on the swine production system and an emerging zoonotic infection that commonly causes adult bacterial meningitis in Southeast Asian countries [1]. The prevalence of antimicrobial resistant *S. suis* has increased worldwide, likely due to the selective pressure of widespread uses of antibiotics in both veterinary and human medicine. The rising trend of antimicrobial resistant *S. suis* signals is an alarming global health problem.

The worldwide antimicrobial resistance data reveal that the resistance prevalence of *S. suis* can be highly variable, depending on the geographical location, management of antibiotic prescription, and health condition [1,8]. Among the *S. suis* strains isolated in this study, the majority were serotypes 2 (25.8%), 8 (8.4%), and 29 (7.6%). These serotypes have been known to colonize the upper respiratory tract of healthy carriers for long periods [7]. Even if *S. suis* is not always targeted, it can be exposed to massive amounts of antibiotics used to eradicate weaning diarrhea, intestinal infections, and respiratory disease in swine production. These may potentially lead to the development of widespread antibiotic resistance [3,4,7].

β-lactams (amoxicillin/clavulanic acid, ampicillin, ceftiofur, ceftriaxone, and penicillin) are still the drug of choice for the treatment of *S. suis* infection; meanwhile, a large body of knowledge shows the growing trend of the isolates resistant to penicillin (0–27%), ampicillin (0.6–23%), and ceftiofur (0–23%) [1,7,8,9]. The findings from this study are consistent with the previous literature. Moreover, this study has demonstrated isolates with a higher rate of penicillin resistance (50.2%). Compared to low penicillin MIC_50_ (≤0.03–0.25 µg/mL) and MIC_90_ (0.25–2 µg/mL) reported from many countries [10,11,12], our study revealed relatively high penicillin MIC_50_ (1.0 µg/mL) and MIC_90_ (>8.0 µg/mL). In addition, it emerged that *S. suis* isolates displayed reduced susceptibility to ampicillin, amoxicillin/clavulanic acid, and cephalosporins.

Variations within PBPs, especially the residues adjacent to or within the three active site motifs: SXXK, SXN and KXG of TPD, are the major resistance mechanism responsible for reduced β-lactams susceptibility in streptococci. The level of resistance is likely increased along with the degree of amino acid changes in PBPs. It has been suggested that changes in PBP2b are responsible for penicillin, PBP2x gene mutations are selected by cefotaxime, while high-level resistance is achieved with additional changes in PBP1a [5]. 

In this study, the analysis of amino acid sequences of *S. suis* PBPs revealed a high degree of genetic diversity among all four PBPs. The numbers of amino acid variations in PBP2a (22 residues) and PBP2b (38 residues) were comparable, while that of PBP1a was high (55 residues), and the greatest numbers of amino acid variations can be found in PBP2x (109 residues). A three-dimensional structure has revealed *S. pneumoniae* PBP2x as the primary target undergoing the amino acid modification under antibiotic pressure [13]. Therefore, the higher probability of β-lactams resistance in *S. suis* might be mediated by the higher numbers of amino acid alterations in PBP2x.

It has been reported that T371A/S substitution of PBP1a ^370^STMK active site motif led to a reorientation of the serine residue, resulting in high-level resistance against PEN and cephalosporins in *S. pneumoniae* isolates [14,15]. In this study, no amino acid substitution at ^370^STMK active site motif of PBP1a was identified; however, two PEN-resistant *S. suis* isolates with MIC ≥ 8 µg/mL harboring the T562S mutation at PBP1a ^561^KTG active site motif together with other mutations was found. The data suggested that the accumulation of PBP1a mutations in the active site motifs and the other regions might be able to elevate the degree of PEN resistance in the *S. suis* population. 

A recent study has reported two human patient-isolated *S. suis* strains with intermediate resistance to PEN, harboring PBP1a P409T, PBP2b T584A, and PBP2b H588Y, found in northern Thailand [16]. This study reported the same triple substitutions found in 122 diseased-pig *S. suis* isolated from central Thailand, of which 109 isolates exhibited PEN-non-susceptibility (12 isolates of PEN-intermediate resistance and 97 isolates of PEN-resistance). The presence of the same triple substitution in *S. suis* strains isolated from different sources and regions suggested widespread prevalence of triple mutant variants of *S. suis* in Thailand.

In this study, the RR analysis revealed the association between PBP1a mutations and reduced β-lactams susceptibility (Figure 3); however, there were some *S. suis* isolates that harbored those mutations without becoming non-susceptible. The association between PBP1a P409T mutation and reduced PEN susceptibility was significant (RR = 2.1); however, there were 18.1% of PEN-susceptible isolates exhibiting a slightly increased PEN MIC value (0.06–0.25 µg/mL) that also harbored the PBP1a P409T mutation. This PBP1a P409T significantly displayed a strong relationship with reduced susceptibility to AMP (RR = 18.2) and AMC (RR = 17.0). Among the PBP1a mutations, the M587S/T substitution was predicted to have the largest impact on reduced susceptibility to AMC (RR = 44.4) and CTX (RR = 2.1); however, some isolates with AMC and CTX susceptibility also carried these mutations. The PBP1a S477D/G likely contributed to the decrease in FUR susceptibility (RR = 17.5), and the coexistence of PBP1a K522E/Q/S and K525Q/R might have a strong relationship with the decrease in susceptibility to fourth generation cephalosporin CPM (RR = 6.4); however, these mutations could also be found in cephalosporin-susceptible isolates. Hence, it could be suggested that the presence of PBP1a mutations alone might not be sufficient to obtain the β-lactams-non-susceptibility, but they could contribute to increasing the MIC level and the stepwise accumulation of several mutations in the PBP1a and other PBPs could eventually lead to β-lactams-resistant phenotype for *S. suis*. 

A few cases of substitutions in PBP2a have been found to contribute to the β-lactams resistance in streptococci. Notably, T411A substitution within the active site serine at ^410^STIK motif has been observed in *S. pneumoniae* isolates [5] and PBP2a T397A substitution has been reported in oxacillin-resistant *S. uberis* from Canada and the UK [17]. To the best of our knowledge, this is the first report of mutations nearby PBP2a ^592^KTG conserved motif, and T584A and H588Y substitutions in PEN-non-susceptible *S. suis* isolated from diseased pigs. In this study, the presence of PBP2a T584A and PBP2a H588Y mutations was also related to increased AMP MIC level. However, due to a relatively low binding affinity between PBP2a and b-lactams, it has been suggested that under drug pressure, the PBP1a mutations are primarily selected before PBP2a becomes a factor in the resistance development [5]. 

Previous studies have shown that the most significant mutations in PBP2b include the T446A substitution in proximity to the ^443^SSN motif displaying a 60% reduction in penicillin affinity in *S. pneumoniae*. Due to the side chain of T446 contributing to stabilizing polar and hydrophobic interactions of residues surrounding the active site, the T446A mutation could perturb structural integrity around the ^443^SSN active site motif [18]. However, this mutation can also be found in PEN susceptible *S. pneumoniae* isolates [19]. In this study, amino acid substitution PBP2b I452A/S/V adjacent to ^451^SSN motif, was observed in both PEN-non-susceptible (90.2%) and PEN-susceptible (40.3%) isolates. Other common PBP2b substitutions, including K479T, D512E, K513E, and T515S were also identified. These mutations have been reported to be related to PEN-resistant *S. suis* strains isolated in the major pig-producing regions, the UK, Canada, and Vietnam [20]. It is likely that these PBP2b substitutions may also play a part in the reduction in PEN susceptibility in *S. suis* strains isolated in Thailand. 

None of the isolates in this study had amino acid changes in the three active site motifs of PBP2a and PBP2b and there was only one mutation, M341I in the active site ^339^STMK of PBP2x. High-level PEN-resistant isolates with MIC of ≥8.0 µg/mL were found in 31 *S. suis* isolates (31/153, 20.3%), of which the PBP2x substitution rate was high. Although M341I substitution in the ^339^STMK conserve motif of PBP2x was not abundant among PEN-non-susceptible *S. suis* isolates, the presence of M341I was strongly associated with high-level PEN-resistant isolates (MIC of ≥8.0 µg/mL). Our results are in agreement with those previously reported by Hu et al. [21], which showed that the alteration of M341I in PBP2x (equivalent to M339 and M342 of *S. pneumoniae* and *S. pyogenes* PBP2x, respectively) was a key mutation responsible for β-lactams resistance in pig-isolated *S. suis* strain R61 (MIC of >4 µg/mL) in China. In addition, *S. pneumoniae* containing T338A together with M339F substitutions caused a distortion of the active site serine (S337), leading to a 4–10-fold reduction in the reaction rate with β-lactams. As a result, *S. pneumoniae* with PBP2x T338A and M339F exhibited high-level resistance against β-lactams [5,22]. It is likely that the M341I substitution in PBP2x could be a PEN-resistant determinant to elevate the high PEN MIC level. 

However, the limitation in this study was that the association of the mutations in TPD of PBBs with PEN and other β-lactams susceptibility was based on the statistical analysis only. While this study revealed mutations in that the PBP were statistically associated with β-lactams-non-susceptible phenotype, several novel mutations were identified from our *S. suis* population. The exact roles of those alterations responsible for β-lactams susceptibility in *S. suis* require further investigation. 

## 4. Materials and Methods

### 4.1. Streptococcus suis Isolates

A total of 225 non-duplicate *S. suis* isolates were recovered from diseased pigs between the years of 2018 to 2020, kindly obtained from Assistant Professor Dr. Pornchalit Assavacheep, Department of Veterinary Medicine, Faculty of Veterinary Science, Chulalongkorn University. Most of them were isolated from lung tissue (81.8%, 184/225), followed by brain tissue (8.0%, 18/225), nasal swab (4.4%, 10/225), and joint drainage (2.2%, 5/225). Other specimen types included blood, spleen tissues, vaginal swab, pleural effusion, and tongue swab, each accounting for ≤1.0% of the isolates (Table S1). The clinical strains were isolated on Columbia blood agar with 5% sheep blood at 37 °C in 5% CO_2_ overnight. The isolates with alpha hemolytic colonies were further identified by conventional biochemical tests [23]. All presumptive *S. suis* isolates were then confirmed to be *S. suis* by the PCR targeting the glutamate dehydrogenase (*gdh*) and the recombination/repair protein (*recN*) genes. In addition, *S. suis* serotypes were well characterized by using the multiplex PCR-based method. If the test strain was negative with all primers, it was classified as non-typeable (NT) as described in Lunha et al. [24].

### 4.2. Antimicrobial Susceptibility Assays

The minimum inhibitory concentrations (MICs) of *S. suis* against penicillin (PEN) and other β-lactams including ampicillin (AMP), amoxicillin/clavulanic acid (AMC), cefuroxime (CXM), ceftiofur (FUR), ceftriaxone (CRO), cefotaxime (CTX), and cefepime (CPM), were determined by the broth-microdilution method, using a commercially prepared Sensititre BOP06F and STP6F microdilution plates (Trek Diagnostic Systems Ltd., East Grinstead, UK), according to the manufacturer’s instructions. *Streptococcus pneumoniae* ATCC 49619 was applied as a quality control strain and the results were within the CLSI defined quality standards. The MIC results were interpreted by using CLSI veterinary breakpoints (CLSI Vet01S, 2020), EUCAST (EUCAST, 2020), and FDA (FDA, 2019), which were previously published by Lunha et al. [24]. The isolates showing intermediate susceptibility and resistance to each agent were classified as *S. suis* with non-susceptible to β-lactams.

### 4.3. DNA Extraction and Whole Genome Sequencing

All isolates were grown overnight on Todd-Hewitt broth (HiMedia, Mumbai, India) at 37 °C in 5% CO_2_, and genomic DNA (gDNA) were extracted by cetyl trimethylammonium bromide (CTAB) method [25] with minor modification. Briefly, cells were collected and resuspended in lysis buffer containing 2.4 mg/mL lysozyme (Sigma Aldrich, St. Louis, MO, USA). Bacterial cells were lysed in 0.5% sodium dodecyl sulphate (SDS) and 0.1 mg/mL proteinase K (Invitrogen). Proteins and polysaccharides were precipitated in 0.5 M NaCl and CTAB/NaCl (10% CTAB, 0.7 M NaCl). DNA purification was carried out by mixing with chloroform-isoamyl alcohol mixture (24:1) to separate contaminants into the organic phase and nucleic acid into the aqueous phase. The aqueous-phase solution was subjected to 0.1 mg/mL RNase A (Sigma Aldrich) treatment and absolute isopropanol was then added to precipitate the nucleic acids. The DNA precipitate was collected and washed with 70% ethanol. The gDNA was dried at 60 °C for 1 h and resuspended in 50 µL of distilled water. The *S. suis* gDNA purification was conducted using DNA Clean & Concentrator kit (Zymo research, Irvine, CA, USA), according to the manufacturer’s instructions. All processed samples had a minimum DNA concentration of 30 ng/μL and OD_260_/OD_280_ in the range of 1.8–2.0 verified by the NanoDrop spectrophotometer (Thermo Fisher Scientific, Fair Lawn, NJ, USA). Samples were submitted to GeneWiz sequencing facility (GeneWiz Inc., Hangzhou, China) and sequenced on the HiSeq Illumina platform (Illumina, San Diego, CA, USA) generating ∼2 GB of 150 bp pair-end sequencing reads with >100X coverage. 

### 4.4. Sequence Analysis

Assemblies and annotations were generated by open software, PATRIC database (Pathosystems Resource Integration Center-https: https://www.patricbrc.org, accessed on 7 November 2021), with default bioinformatics pipeline. Adapter and low-quality sequences were trimmed from the generated read sequences using Trim Galore with a sliding window quality cutoff of Q20. The de novo assembly was performed using Unicycler genome assembler with minimum contigs of 300 bp produced. Draft genomes were annotated using the RAST tool kit. The poor sequencing quality genomes with a large number of contigs (>1000), N50 values of <10,000 bp or that which were inconsistent with an *S. suis* genomes in the GenBank database were excluded from the analysis. 

The full-length recombination/repair protein (*recN*) gene was extracted from the annotated genomes. The test isolates were confirmed as *S. suis* by having a ≥95% nucleotide identity to *S. suis*-specific *recN* sequence of the reference genome, *S. suis* P1/7, GenBank accession no. AM946016.1. The average nucleotide identity (ANI) between the isolates and the reference genome was also calculated by FastANI v1.3 with cut-off of ≥94% identity [26]. *S. suis* serotype was confirmed using gene content of capsular polysaccharide (*cps*) loci. Two serotype pairs with similar sequence (2 or ½) and (1 or 14) were differentiated using the *cpsK* gene, a single-nucleotide (G→C/T) substitution at nucleotide position 483 in the case of serotype ½ and 1 [27].

The sequences of *pbp* genes, *pbp1a*, *2a*, *2b*, and *2x*, were extracted from the studied isolates. The single nucleotide polymorphisms associated with amino acid substitution were identified by the multiple sequence alignment against the reference strain *S. suis* P1/7 (GenBank accession no. AM946016.1) using the ClastalW in MEGA-X [28]. 

### 4.5. Statistical Analysis

Data analysis was performed using the STATA (version 14.0; Stata Corporation, College Station, TX, USA). The Pearson’s chi-square test was used to compare the prevalence of various PBPs amino acid substitutions in different β-lactams susceptibility. The relationship between the specific PBPs alterations and β-lactams susceptibility phenotypes was also calculated, and the association was reported as a risk ratio (RR) [29]. A RR of >1 was considered as the increasing probability of the co-occurrence of the genotype being studied with the measured phenotype (positive association), while RR of <1 was considered as the decreasing probability of the co-occurrence of the genotype being studied with the measured phenotype (negative association). A candidate alteration with *p*-value < 0.05 was considered to be independently associated with reduce susceptibility of the β-lactam antibiotics and thus a likely causal variant for resistance. 

## 5. Conclusions

This study has found widespread evidence for increased MIC of β-lactams in the zoonosis pathogen *S. suis* isolated from diseased pigs. A high genetic diversity was observed along the TPD of all four PBPs. The majority of isolates with an increased MIC value for PEN were found to possess the substitutions of PBP1a (P409T) and PBP2a (T584A and H588Y). An additional M341I mutation in ^339^STMK active site motif of PBP2x led to a higher level of PEN resistance (MIC of ≥8.0 µg/mL). The AMP-non-susceptibility was predicted to be related to the mutations of PBP1a (P409T), PBP2a (T584A and H588Y), PBP2b (T625R), and PBP2x (T467S). The mutations of PBP1a (M587S/T), PBP2a (M433T), PBP2b (I428L), and PBP2x (Q405E/K/L) potentially play significant roles in the decrease in AMC susceptibility. Among the cephalosporins, the major mutations associated with non-susceptible cephalosporins were observed against FUR including the mutations of PBP1a (S477D/G), PBP2a (E549Q and A568S), PBP2b (T625R), and PBP2x (Q453H). Given the high degree of PBP mutants in *S. suis* observed, our study suggests the potential for *S. suis* to accumulate more PBP mutations with continued selection pressure and become increasingly more resistance to β-lactams, which could severely threaten the swine production and reduced treatment efficacy in human. 

## Data Availability

Not applicable.

## Supplementary Materials

The following supporting information can be downloaded at: https://www.mdpi.com/article/10.3390/antibiotics12010158/s1, Table S1: Distribution of *S. suis* serotypes in different sources of specimens; Table S2: Minimum inhibitory concentration (MIC) values distribution and resistance rates of *S. suis* isolates; Table S3a: Deduced amino acid alterations in transpeptidase domain (TPD) of penicillin-binding protein PBP1a; Table S3b: Deduced amino acid alterations in transpeptidase domain (TPD) of penicillin-binding protein PBP2a; Table S3c: Deduced amino acid alterations in transpeptidase domain (TPD) of penicillin-binding protein PBP2b; Table S3d: Deduced amino acid alterations in transpeptidase domain (TPD) of penicillin-binding protein PBP2x; Table S4: Amino acid substitutions within the transpeptidase domain (TPD) of penicillin binding proteins (PBPs) and number of *S. suis* isolates with each variant, and the percent different of variant between the isolates being non-susceptible (NS) and susceptible (S); Table S5: The association between PBPs amino acid substitutions and reduced susceptibility to β-lactams.
